# Psycho-education programme for temporomandibular disorders: a pilot study

**DOI:** 10.1186/1477-5751-6-4

**Published:** 2007-03-23

**Authors:** Waseem Jerjes, Geir Madland, Charlotte Feinmann, Mohammed El Maaytah, Mahesh Kumar, Colin Hopper, Tahwinder Upile, Stanton Newman

**Affiliations:** 1 Unit of Oral & Maxillofacial Surgery, Division of Maxillofacial, Diagnostic, Medical and Surgical Sciences, Eastman Dental Institute & University College London, London, UK; 2 Department of Oral & Maxillofacial Surgery/Head & Neck Unit, University College London Hospitals, London, UK; 3 National Medical Laser Centre, Department of Surgery, Royal Free & University College Medical School, London, UK; 4 Unit of Oral Medicine & Special Needs Dentistry, Oral Medicine and Special Needs Dentistry, Division of Maxillofacial, Diagnostic, Medical and Surgical Sciences, Eastman Dental Institute, London, UK; 5 Centre for Behavioural and Social Sciences in Medicine, Royal Free & University College Medical School, London, UK

## Abstract

**Background:**

Temporomandibular disorders (TMDs) are by far the most predominant condition affecting the temporomandibular joint (TMJ), however many patients have mild self-limiting symptoms and should not be referred for specialist care.

The aim of this pilot study was to develop a simple, cost-effective management programme for TMDs using CD-ROM. 41 patients (age 18–70) participated in this study, patients were divided into three groups: the 1st group were involved in an attention placebo CD-ROM (contain anatomical information about the temporomandibular system), the 2nd group received information on CD-ROM designed to increase their control and self efficacy, while the 3rd group received the same programme of the 2nd group added to it an introduction to self-relaxing techniques followed by audio tape of progressive muscle relaxation exercises. Each of the groups was asked to complete a number of questionnaires on the day of initial consultation and six weeks afterwards.

**Results:**

The two experimental groups (2nd & 3rd) were equally effective in reducing pain, disability and distress, and both were more effective than the attention placebo group (1st), however the experimental groups appeared to have improved at follow-up relative to the placebo-group in terms of disability, pain and depressed mood.

**Conclusion:**

This pilot study demonstrates the feasibility and acceptability of the design. A full, randomized, controlled trial is required to confirm the efficacy of the interventions developed here.

## Background

Temporomandibular disorders comprise the most common non-infective pain condition of the orofacial region [1]. It is by far the most predominant condition described that affects the temporomandibular system; other conditions include arthritis, arthrosis, arthralgia and disc displacement [1,2].

TMDs is clinically characterized by pain in the temporomandibular region or in the muscles of mastication, pain radiating (behind the eyes, in the face, shoulder, neck and/or the back), headaches, earaches or tinnitus, jaw clicking, locking or deviation, limited jaw opening, clenching or grinding of the teeth, dizziness and sensitivity of the teeth without the presence of an oral disease [2,3].

TMDs signs were also identified in asymptomatic individuals [4-6]; a cross-sectional population-based survey [7] was conducted in the United Kingdom, involving 2504 participants (participation rate 74%), of whom 646 (26%) reported orofacial pain. Overall, 424 (79% adjusted participation rate) of those individuals participated at the four-year follow-up, of whom 229 (54%) reported orofacial pain and 195 (46%) did not report such pain. Persistent orofacial pain was associated with females, older age, psychological distress, widespread body pain, and taking medication for orofacial pain at baseline.

De Kanter et al. [8] carried out a nationwide survey of oral conditions, treatment needs, and attitudes toward dental health care in Dutch adults. They found that a total of 21.5% of the Dutch adult population reported dysfunction, but 85% of these perceived no need for treatment. With most of the remaining 15% either seeking or intending to seek treatment (or having had it before), a figure of 3.1% can be used to summarize the actual level of treatment need for TMDs in the Dutch adult population.

The aetiology of TMDs is both of structural and psychological concepts. Structural concepts are classified as conditions related to the temporomandibular joint (TMJ) itself (functional, structural, morphopathological; i.e. micro-/macro-trauma), conditions related to the muscles of mastication (muscle spasm i.e. parafunctional habits) or occlusal factors (i.e. bruxism); recent studies had shown that occlusal factors were not found to be directly involved with TMDs; nevertheless they could contribute with other factors or aggravate an existing condition [8-11].

Psychological theories includes stressful life events [12,13], post-traumatic stress disorder [14], psychiatric illness (anxiety and depression) [15,16], somatoform disorders [17] and personality disorders (i.e. obsessive-compulsive disorder), hypochondriasis, paranoia, schizophrenia [14,18].

Clinicians may obfuscate the problem by concentrating on examination of the physical component (location and severity of pain, TMJ and related muscles) and disregard the psycho-social and behavioral factors. The introduction of Research Diagnostic Criteria (RDC), by Dworkin and LeResche [19] at the University of Washington, for the TMDs established a proper diagnostic criterion for this condition; this dual-axis system may be superior to other instruments, since it can be used to classify and quantify both physical and psychosocial components of the TMDs.

The aim of this pilot study was to develop a simple, cost-effective and evidence-based management programme for TMDs, using CD-ROM. A comparison group received adjunctive relaxation training, known to be effective in the management of this disorder.

## Methods

41 TMDs patients, awaiting for consultation appointment, took part in this pilot study; patients were then divided into three groups from which some received psycho-educational programmes and the other received placebo programmes; these programmes were designed at the Eastman Dental Institute, University College London. The multi-central trial protocol was approved by the joint UCL/UCLH committees of the ethics for human research.

Inclusion criteria included English speakers with age of 18–70 years old and should satisfy the research diagnostic criteria (RDC/TMD) [9]. Patients were recruited from a variety of Oral and Maxillofacial Surgery (OMFS) Departments in London.

An information sheet explaining the aim of our study in simple non-scientific terms was given to each of the patients. Each patient was asked to sign a consent form. All patients were invited to complete a number of questionnaires at the time of the initial appointment (Table 1).

**Table 1:** Questionnaires used in our pilot study

Questionnaires	
OHIP	Oral Health Impact Profile
VAS	Visual Analogue Scale
PRI	Pain Rating Index
STAI	State-Trait Anger Expression Inventory
BDI	Beck Depression Inventory
IPQ	Illness Perceptions Questionnaire
CSQ	Coping Strategies Questionnaire
MHLC	Multidimensional Health Locus of Control scale
SES	Self-Efficacy Scale

Information collected from every patient included: age, gender, pain duration (months), frequency of pain (days with pain during preceding month) and jaw opening. Additional information included information on joint sounds, myalgia, arthralgia, the number of healthcare visits over the past 6 months regarding their temporomandibular joint (TMJ) problems and information about their analgesic or prophylactic medications.

12 of those patients were subjected to an attention placebo CD-ROM comprising anatomical information on the TMJ and the muscles of mastication group 1), 15 patients received information on CD-ROM designed to empower them (increasing their control and self-efficacy) (group 2); the rest of the patients received group 2 treatment with an additional introduction to self-relaxation techniques, followed by an audiotape of progressive muscle relaxation exercises to be dispatched from a central source (independent of the researcher) (group 3).

Patients were also asked to complete a number of questionnaires (OHIP, VAS, VRI, STAI and BDI) six weeks following the educational programme; which would enable us to conduct correlation analysis. Patients were also asked to complete feedback questionnaires {Pain Stages of Change Questionnaire (PSOCQ)}.

### Technical report

The voiceover for the CD was edited and manipulated using CoolEdit 95 (Syntrillium Software Corporation, 1995). Original photographs were captured with digital cameras (Nikon CoolPix 950 and Kodak CD 215). Other photographs were sourced from the Photo-Objects Collection (Hemera Technologies Inc., 1998).

Images were manipulated and edited in Paintshop Pro version 5 (JASC Software Inc., 1998) and Adobe Photoshop version 5.5 (Adobe systems Inc., 1999). The elements were brought together using Macromedia Flash version 4 (Macromedia Inc., 1999). The Flash movie settings were 100% image quality and 22 MHz mono sound. Given the technology used, the programme can, with additional editing, be reconfigured to stream across the Internet rather than run from a CD. The resulting CD requires a PC running Windows 95, 98 or 2000 with pointing device, sound card, 64 MB RAM and an 8-speed CD player.

### Statistical analysis

The Chi-squared statistic was used to test for differences in clinical examinations as well as questionnaires and checklists between the three groups at the first specialist consultation and at the follow up consultation after 6 weeks. The same test was used also to look for differences between the groups.

## Results

### Population

41 patients participated in this study, 1st group comprised 12 patients, 2nd group 15 patients, and the rest of patients represented group 3; mean age was 37 years old; female overrepresentation reached a mean of 89%.

Patients in group 1 represented those with the highest duration of pain that reached an average of 95 months; as well as the frequency of pain (days per month); the same group had an increase in deviation on opening and assisted opening when compared to the other groups (Table 2).

**Table 2:** Comparing the 3 groups at the initial consultation (I)

	All patients	Group 1	Group 2	Group 3
No. of patients	41	12	15	14
Age (years)	37 ± 13	37 ± 13	36 ± 12	42 ± 16
Sex (% females)	89%	92%	93%	79%
No. of years in education	15 ± 4	13 ± 5	17 ± 4	15 ± 3
Duration of pain (months)	53 ± 67	95 ± 111	45 ± 42	46 ± 21
Frequency (days per month)	22 ± 10	26 ± 7	21 ± 10	19 ± 12
Deviation on opening (%)	17%	33%	20%	8%
Ass.opening + overbite (mm)	38 ± 7	40 ± 6	38 ± 8	36 ± 7

### Clinical signs

Joint sound and arthralgia were found to predominate in the first group; while myalgia was more common in the second group. Patients in this study required health care services four times in six months. 40% of patients in group 2 required analgesic medications; while 17% of group 1 required prophylactic medication (Table 3).

**Table 3:** Comparing the 3 groups at the initial consultation (II)

	All patients	Group 1	Group 2	Group 3
Joint sounds (%)	55%	75%	53%	43%
Myalgia (%)	28 %	25%	27%	21%
Arthralgia (%)	32%	42%	20%	29%
Health care visits (past 6 months)	4 ± 5	4 ± 8	2 ± 3	3 ± 3
Analgesic medication (%)	40%	25%	40%	21%
Prophylactic medication (%)	9%	17%	7%	0

### Results

Patients in group 1 showed higher scores of OHIP, VAS, VRI, BDI and IPQ (consequence and performance) when compared to group 2, which showed high scores in STAI and IPQ (performance and cure); while group 3 scores were high in the IPQ (cyclic, puzzling and personal control) (Table 4). Group 1 scores were high in CSQ (diverting attention, increasing behavioral activity, praying/hoping, catastrophising and control) and MHLC (external [powerful others]); Group 2 showed high scores in CSQ (reinterpreting sensations), MHLC (internal and external [chance]) and SES, while group 3 scored higher only in the ignoring sensations and coping self-statements of the CSQ (Table 5).

**Table 4:** Comparing the 3 groups at the initial consultation (III)

	All patients	Group 1	Group 2	Group 3
**OHIP**: Disability (0–56)	19.0 ± 10.0	26.0 ± 11.0	19.0 ± 10.0	17.0 ± 7.0
**VAS**: Past pain (1–100 mm)	57.0 ± 21.0	63.0 ± 16.0	59.0 ± 25.0	46.0 ± 21.0
**PRI**: Past pain (0–5)	2.5 ± 1.1	2.8 ± 1.0	2.7 ± 1.1	1.8 ± 1.1
**STAI**: Anxious mood (0–18)	7.3 ± 4.2	6.4 ± 4.6	7.5 ± 4.2	5.9 ± 2.8
**BDI**: Depressed mood (0–36)	12.5 ± 11.1	13.6 ± 11.5	11.2 ± 12.1	11.4 ± 9.2
				
**IPQ**: Consequence (11–55)	26.9 ± 7.4	30.0 ± 8.9	24.7 ± 7.2	25.2 ± 4.1
Performance (3–15)	8.9 ± 2.6	8.6 ± 1.2	8.6 ± 2.9	8.4 ± 2.5
Cyclic (4–20)	12.3 ± 3.4	12.6 ± 2.6	12.0 ± 4.3	12.7 ± 3.2
Puzzling (1–5)	3.5 ± 1.0	3.4 ± 0.9	3.1 ± 0.9	3.7 ± 1.0
Cure (5–25)	16.7 ± 2.4	16.7 ± 2.6	17.1 ± 2.9	16.7 ± 1.0
Personal control (7–35)	22.0 ± 3.6	22.0 ± 2.3	22.1 ± 3.0	22.9 ± 3.7

**Table 5:** Comparing the 3 groups at the initial consultation (IV)

	All patients	Group 1	Group 2	Group 3
**CSQ**: Diverting attention (0–36)	6.1 ± 5.9	7.4 ± 4.3	5.6 ± 5.8	6.1 ± 7.8
Increase behavioural act. (0–36)	12.3 ± 10.1	14.0 ± 10.5	11.5 ± 10.8	11.9 ± 10.5
Ignoring sensations (0–36)	6.2 ± 6.9	5.4 ± 5.6	5.6 ± 6.9	7.0 ± 8.4
Reinterpreting sensations (0–36)	18.9 ± 8.5	19.4 ± 6.4	20.0 ± 7.0	17.4 ± 11.8
Coping self-statements (0–36)	14.4 ± 8.0	13.7 ± 7.8	13.7 ± 6.5	15.6 ± 10.4
Praying/hoping (0–36)	9.6 ± 7.8	12.5 ± 7.9	8.9 ± 9.0	7.1 ± 5.6
Catastrophising (0–36)	8.9 ± 8.7	10.9 ± 9.4	9.9 ± 9.9	4.4 ± 3.8
Control (0–12)	3.9 ± 3.5	4.7 ± 2.9	3.7 ± 4.0	3.8 ± 3.5
				
**MHLC**: Internal (6–36)	22.7 ± 4.9	22.7 ± 4.8	24.6 ± 5.2	22.1 ± 3.0
External (Chance) (6–36)	17.7 ± 5.5	17.9 ± 5.3	18.6 ± 6.5	16.2 ± 5.0
External (powerful others) (6–36)	14.4 ± 6.0	16.6 ± 5.2	12.8 ± 5.2	15.4 ± 7.1
				
**SES**: self-efficacy (0–30)	20.1 ± 5.4	18.8 ± 3.5	20.8 ± 5.4	20.6 ± 4.9

Correlation analysis was conducted for the entire group (Table 6). Significant correlations (p < 0.01) were found for the principal dependent variables. Anxious mood (STAI) correlated with depressed mood (BDI, 0.645) and catastrophising (CSQ, 0.413). Depressed mood also correlated with disability (OHIP, 0.592) and consequence (IPQ, 0.413).

**Table 6:** Comparing the 3 groups 6 weeks after the initial consultation

	All patients	Group 1	Group 2	Group 3
	37 (90%)	10 (83%)	15(100%)	12 (86%)
**OHIP**: disability (0–56)	16.8 ± 11.0	21.3 ± 12.0	15.4 ± 12.2	14.8 ± 7.7
**VAS**: past pain (mm)	41.0 ± 29.0	52.0 ± 35.0	45.0 ± 30.0	27.0 ± 18.0
**PRI**: past pain (0–5)	1.8 ± 1.2	2.3 ± 1.4	1.9 ± 1.2	1.3 ± 0.6
**STAI**: anxious mood (0–18)	6.8 ± 5.0	7.6 ± 6.4	7.1 ± 5.2	5.9 ± 3.4
**BDI**: depressed mood (0–63)	9.3 ± 9.9	10.9 ± 8.1	8.5 ± 12.0	8.8 ± 9.3
				
**PSOCQ**:				
Pre-contemplation (7–35)	19.7 ± 6.2	22.2 ± 7.0	18.1 ± 4.7	19.4 ± 6.9
Contemplation (10–50)	33.0 ± 6.9	35.2 ± 4.0	33.5 ± 6.7	30.6 ± 8.7
Action (6–30)	18.3 ± 5.1	17.3 ± 4.6	18.9 ± 5.5	18.6 ± 5.1
Maintenance (7–35)	22.4 ± 5.6	20.7 ± 5.1	23.1 ± 5.3	22.9 ± 6.3

Disability also correlated with pain (VRI, 0.459), consequence (IPQ, 0.588), diverting attention (CSQ, 0.440) and catastrophising (0.432). Pain over the past month (verbal rating) correlated with pain (VAS, 0.491), frequency (0.436), disability (0.459), consequence (0.551) and catastrophising (0.675).

Monthly frequency of pain also correlated with belief in the performance (IPQ, 0.468) and negative with cyclical nature (IPQ, 0.516) of the pain. Health care visits correlated negatively with a belief in care (IPQ, 0.439).

### Feedback

Feedback questionnaires were considered for the entire group together so that blinding of the investigator to group was not compromised. Feedback about the programme was generally positive (Intervention Value Scale), with patients appreciating the decision of cause and treatment options, and the self-management advice, whilst some expressed understandable disappointment at the lack of definitive cure.

Patients were asked to rate the programme on Likert-type scales of 1 to 5 for "bad" to "excellent", resulting in means (± SD) of about 4.1 ± 0.9 for overall usefulness; 4.1 ± 0.9 for usefulness of information; and 3.8 ± 1.0 for usefulness of advice. Similar scales were used to rate the programme in relation to a written leaflet (4.2 ± 0.8) and to a personal consultation (3.3 ± 1.1). 78% of the patients considered the length of the programme to be appropriate, and 85% felt they were likely to practice any self-help suggestions from the programme.

## Discussion

The literature on the topic of education programmes for chronic pain is sparse, despite their importance in the care of patients with, for example, fibromyalgia [20]. Arthritis Self-Management Programme (ASMP) was found to produce long terms benefits in terms of reduced pain ratings and arthritisrelated physician visits [21].

A pilot study conducted by Tutty et al. [22] on telephone counseling as an adjunct to antidepressant treatment in the primary care system. 28 adult primary care patients starting antidepressant treatment (telephone counseling group) was compared with 94 patients receiving usual care (control group). Results have shown that telephone counseling patients showed significantly lower depressive symptoms than did control group patients at 3-month and 6-month follow-up.

Other studies have established benefits from telephone and mail-delivered self-assessment programmes [23-27], for example arthritis [23,24]. In a randomized controlled trial of education and physical training for women with fibromyalgia, of which TMDs may be a variant, the education programme was found to enhance self-efficacy, although changes in disability and distress were more modest [28]. These results are encouraging when one considers the potential effect of enhanced self-efficacy on perceived control over time. A Canadian group of mixed idiopathic chronic pain patients, assigned to a community based psycho-education programme modified from the Arthritis Self-Management Programme (ASMP), made significant short term improvements in pain, dependency, vitality, aspects of role functioning, life satisfaction and in self-efficacy and resourcefulness, compared to a waiting-list control group [25].

Although education programmes are advocated for TMDs, they have not been adequately evaluated. Relaxation training, however, has shown to be equally effective as conventional occlusal splint therapy [26] and benefits may be longer lasting [27].

TMDs patient improvement after conservative dental treatment was modestly associated with changes in beliefs and coping with and without a brief cognitive-behavior intervention [28], suggesting that such changes may accompany simpler treatments. A single trial of a psycho-educational group intervention showed modest but enduring reduction in TMDs-related interference compared to usual treatment [29].

In this study, it was hypothesized that the two experimental groups (2nd & 3rd) would be equally effective in reducing, pain disability and distress, and both are more effective than the attention placebo group. Primary outcome improvements were expected to be associated with the modest ameliorations in pain related cognitions, including self-efficacy.

The experimental groups (2nd & 3rd) appeared to have improved at follow-up relative to the placebo-group in terms of disability, pain and depressed mood. However, it should be noted that the groups, although randomly selected, did in fact differ at the outset, and these trends must be discounted.

The ultimate goal is an interventions specifically aimed at ameliorating cognitions and behavior in TMDs, in order to reduce the associated pain, disability and distress.

## Conclusion

This pilot study demonstrates the feasibility and acceptability of the design. A full, randomized, controlled trial is required to confirm the efficacy of the interventions developed here.

## Competing interests

The author(s) declare that they have no competing interests.

## Authors' contributions

**WJ**: carried out the literature research, manuscript preparation, and manuscript review

**GM**: designed the study, carried out the literature research, clinical study and statistical analysis

**CF**: designed the study, carried out the literature research, clinical study, statistical analysis and manuscript preparation

**ME**: carried out the literature research, manuscript preparation, and manuscript review

**MK**: carried out the literature research, manuscript preparation, and manuscript review

**CH**: carried out the literature research, manuscript preparation, and manuscript review

**TU**: carried out the literature research, manuscript preparation, and manuscript review

**SN**: designed the study, carried out the literature research, clinical study, statistical analysis and manuscript preparation
